# Исследование фотопериодической динамики содержания периферического дофамина в сопоставлении с тиреоидным профилем у различных групп мужчин Европейского Севера

**DOI:** 10.14341/probl13286

**Published:** 2023-06-06

**Authors:** Е. В. Типисова, В. Н. Зябишева, В. А. Аликина, А. Э. Елфимова, И. Н. Молодовская

**Affiliations:** Федеральный исследовательский центр комплексного изучения Арктики имени академика Н.П. Лавёрова Уральского отделения Российской академии наук; Федеральный исследовательский центр комплексного изучения Арктики имени академика Н.П. Лавёрова Уральского отделения Российской академии наук; Федеральный исследовательский центр комплексного изучения Арктики имени академика Н.П. Лавёрова Уральского отделения Российской академии наук; Федеральный исследовательский центр комплексного изучения Арктики имени академика Н.П. Лавёрова Уральского отделения Российской академии наук; Федеральный исследовательский центр комплексного изучения Арктики имени академика Н.П. Лавёрова Уральского отделения Российской академии наук

**Keywords:** мужчины, Россия, сезоны, фотопериод, тиреоидные гормоны, дофамин, кочующее население

## Abstract

**ОБОСНОВАНИЕ:**

ОБОСНОВАНИЕ. Знание физиологических механизмов адаптации, возникающих в ответ на изменение фотопериодов, особенно актуально для жителей Европейского Севера. В литературе практически отсутствуют сведения о фотопериодической динамике уровня дофамина в крови, несмотря на его значимую роль в регуляции деятельности организма. В то же время, известно о взаимном модулирующем эффекте дофаминергической и тиреоидной систем.

**ЦЕЛЬ:**

ЦЕЛЬ. Показать соотношение уровня дофамина и содержания гормонов, белков и аутоантител системы гипофиз – щитовидная железа в крови с учетом фотопериода года у практически здоровых лиц, проживающих на территории Европейского Севера.

**МАТЕРИАЛЫ И МЕТОДЫ:**

МАТЕРИАЛЫ И МЕТОДЫ. Обследовалось 20 мужчин (80 проб)  − практически здоровое мужское население г. Архангельска в различные фотопериоды года: увеличение продолжительности светового дня (март), его максимальная продолжительность (июнь), уменьшение (сентябрь),  минимальная продолжительность (декабрь). Жители поселков и кочующее аборигенное население (100 человек) обследовались в течение 2-х фотопериодов года – март и декабрь. В сыворотке крови определяли уровни йодтиронинов, ТТГ, ТГ, антител к ТПО, антител к ТГ, в плазме – уровень дофамина с использованием методов ИФА.

**РЕЗУЛЬТАТЫ:**

РЕЗУЛЬТАТЫ. У жителей г. Архангельска в июне по сравнению с декабрем выше содержание дофамина в крови (0,502 и 0,365 и нмоль/л, p=0,01), уровней Т3 (1,09 и 0,94 нмоль/л, p=0,003), Т4 (113,45 и 99,03 нмоль/л, p=0,0002). В сентябре по сравнению с июнем регистрировали снижение дофамина (0,235 нмоль/л, p=0,0003), Т3 (0,92 нмоль/л, p=0,004) при повышении ИПК (Т4/Т3) с 106,54 до 117,89 усл.ед. (p=0,006). У кочующего аборигенного населения в марте по сравнению с декабрем зарегистрирована тенденция к более высокому содержанию дофамина (0,00 и 0,394 нмоль/л, p=0,07) при снижении св. Т4 (15,20 и 13,90, p=0,015), ИпПК (св. Т4/св. Т3) с 3,13 до 2,28 усл.ед. (p=0,006). В декабре у 67% кочующего населения регистрируются недетектируемые значения дофамина (0 нмоль/л) и у 22% – сверхнормативные значения дофамина, в марте у 27% – сверхнормативные значения.

**ЗАКЛЮЧЕНИЕ:**

ЗАКЛЮЧЕНИЕ. Показаны однонаправленные изменения в крови уровней дофамина и активности щитовидной железы у мужчин Европейского Севера с их снижением в периоды уменьшения и минимальной продолжительности светового дня и повышением - в периоды нарастания и максимальной длительности светового дня.

## Abstract (en)

**BACKGROUND:**

BACKGROUND: Knowledge of the physiological mechanisms of adaptation arising in response to changes in photoperiods is especially important for residents of the European North. In the literature, there is practically no information about photoperiodic dynamics of serum dopamine level, despite its significant role in the regulation of the body’s activity. The mutual modulating effect of the dopaminergic and thyroid systems is known.

**AIM:**

AIM: To show the ratio of dopamine levels and the content of hormones, protines and autoantibodies of the thyroid system, taking into account photoperiod of the year, in practically healthy populations of the European North.

**MATERIALS AND METHODS:**

MATERIALS AND METHODS: Healthy male population (20 men) of Arkhangelsk was examined in various photoperiods of the year (80 samples): an increase in the length of daylight hours (March), its maximum duration (June), a decrease (September), and a minimum duration (December). The inhabitants of the settlements and the nomadic aboriginal population (100 men) were examined during 2 photoperiods of the year — March and December. The serum levels of iodothyronines, TSH, TG, antibodies to TPO, antibodies to TG and plasma level of dopamine were determined using ELISA methods.

**RESULTS:**

RESULTS: Residents of Arkhangelsk in June compared to December have higher levels of dopamine (0.502 and 0.365 nmol/l, p=0.01), T3 (1.09 and 0.94 nmol/l, p=0.003), T4 (113.45 and 99.03 nmol/l, p=0.0002). In September, compared with June, a decrease in dopamine (0.235 nmol/l, p=0.0003), T3 (0.92 nmol/l, p=0.004) was recorded with an increase in T4/T3 ratio from 106.54 to 117.89 units (p=0.006). The nomadic aboriginal population in March compared with December showed a tendency to a higher content of dopamine (0.00 and 0.394 nmol/l, p=0.07) with the decrease in fT4 (15.20 and  13.90, p=0.015), fT4/fT3 ratio from 3.13 to 2.28 units (p=0.006). In December, 67% of nomadic population had undetectable dopamine values (0 nmol/l) and 22% — excess dopamine values, in March 27% — excess values.

**CONCLUSION:**

CONCLUSION: Unidirectional changes in dopamine and thyroid activity in men of the European North were shown with their decrease during periods of decrease and minimum daylight hours and an increase during periods of increase and maximum daylight hours.

## ОБОСНОВАНИЕ

Проблема изучения фотопериодических реакций организма человека, что особенно актуально для северных широт, не теряет своей актуальности в современном мире. Знание фундаментальных основ физиологических механизмов, возникающих в ответ на изменение освещенности, необходимо для учета состояния различных звеньев организма и своевременного проведения необходимых диагностических, профилактических или лечебных мероприятий. В настоящее время хорошо изучены циркадные ритмы живых организмов. Подробно описаны механизмы влияния освещенности на различные системы организма, в том числе на эндокринную и симпатоадреналовую [1–3]. Окологодовые (сезонные) ритмы начали изучать гораздо позже, и сведений о влиянии мелатонина на тиреоидную ось в контексте годовой изменчивости не так много [1]. Большинство исследований сезонной изменчивости симпатоадреналовой системы связано с изучением содержания норадреналина как одного из маркеров ее активности [4–6], либо вариабельности сердечного ритма [7] или изменения показателей артериального давления [8], а также риска развития гипертонических кризов [9]. Данные, касающиеся ингибирующего влияния мелатонина на высвобождение дофамина, также крайне ограничены [10]. В то же время сведений о сезонных изменениях уровней гормонов щитовидной железы, которые в северных широтах в большой степени связаны с контрастной фотопериодикой, достаточно много [11–12]. Существуют доказательства как ингибирующего, так и взаимного стимулирующего действия дофаминергической и гипоталамо-гипофизарно-тиреоидной систем [13–14], однако сочетанное изменение их активности в динамике фотопериодов года в доступной литературе не встречается. Особый интерес представляет изучение этих особенностей у различных групп населения Европейского Севера, отличающихся либо генетически (европеоидное и аборигенное население), либо образом жизни (оседлое и кочующее аборигенное население).

## ЦЕЛЬ ИССЛЕДОВАНИЯ

Показать соотношение уровня дофамина и содержания гормонов, белков системы «гипофиз — щитовидная железа» и аутоантител к антигенам щитовидной железы в крови с учетом фотопериода года у практически здоровых лиц, проживающих на территории Европейского Севера.

## МАТЕРИАЛЫ И МЕТОДЫ

## Место и время проведения исследования

Место проведения и время исследования

Обследование проводили в г. Архангельске (64°32′24″ с.ш., март, июнь, сентябрь, декабрь 2018 г.), а также в ходе экспедиций в селе Несь Заполярного района Ненецкого автономного округа (НАО) (66°36′06″ с.ш., декабрь 2010 г., 2011 г.), в МО Совполье Мезенского района Архангельской области (65°17′35″ с.ш., март 2012 г.), МО Сояна Мезенского района Архангельской области (65°46′45″ с.ш., март 2013 г.). Сбор материала осуществлялся в различные фотопериоды года: период увеличения продолжительности светового дня, его максимальной продолжительности, уменьшения и минимальной продолжительности светового дня. Критериями выбора месяца обследования были периоды весеннего (март) и осеннего (сентябрь) равноденствия, а также летнего (июнь) и зимнего (декабрь) солнцестояния.

## Изучаемые популяции (одна или несколько)

Изучались 4 популяции постоянных жителей Европейского Севера мужского пола: местные европеоидные жители г. Архангельска (городские жители), постоянные жители поселков (местные европеоидные жители, оседлое аборигенное население), а также кочующее близ поселков аборигенное население.

Популяция «местные жители г. Архангельска»

Критерии включения: мужчины 22–45 лет (молодой возраст по критериям ВОЗ), родившиеся и постоянно проживающие на Севере, европеоидное население.

Популяция «местные жители поселков»

Критерии включения: мужчины 22–59 лет (молодой и средний возраст по критериям ВОЗ), родившиеся и постоянно проживающие на Севере, европеоидное население.

Популяция «оседлое аборигенное население»

Критерии включения: мужчины 22–59 лет (молодой и средний возраст по критериям ВОЗ), ненцы, коми, проживающие в поселках.

Популяция «кочующее аборигенное население»

Критерии включения: мужчины 22–59 лет (молодой и средний возраст по критериям ВОЗ), ненцы, коми, кочующие в периоды обследования близ поселков.

Критерии включения: к группе «практически здоровые лица» относили людей, не состоящих на диспансерном учете у эндокринолога и кардиолога и не имеющих на настоящий момент жалоб со стороны здоровья.

Критерии исключения: лица, находящиеся на учете у эндокринолога, кардиолога, имеющие обострение хронических или наличие острых заболеваний, а также наличие стрессовых ситуаций и употреблявшие алкоголь за 2–3 дня до исследования. К стрессовым ситуациям относили психоэмоциональное напряжение, возникающее на фоне различных семейных, рабочих, общественных событий.

## Способ формирования выборки из изучаемой популяции (или нескольких выборок из нескольких изучаемых популяций)

Способ формирования выборки — вероятностная стратифицированная выборка с распределением по подгруппам в зависимости от характеристик, значимых для исследования.

## Дизайн исследования

Одноцентровое обсервационное исследование. В подгруппе «местные жители г. Архангельска» проводилось динамическое проспективное (в течение года в каждый из сезонов) одновыборочное сравнительное исследование. В подгруппах «местные жители поселков», «оседлое аборигенное население», «кочующее аборигенное население» — одномоментное одновыборочное сравнительное.

## Описание медицинского вмешательства (для интервенционных исследований)

Участники исследования заполняли информированное согласие на исследование, проходили анкетирование, а также измерение антропологических данных, параметров сердечно-сосудистой системы, осуществлялся забор венозной крови медицинским персоналом в утренние часы натощак.

## Методы

Для формирования групп, обследованных с учетом критериев включения и исключения, проводилось анкетирование, а также измерение массы тела, роста, систолического и диастолического артериального давления, частоты сердечных сокращений. У лиц, соответствующих критериям отбора, проводили забор венозной крови.

Уровни гормонов определяли методом иммуноферментного in vitro анализа (ИФА) на планшетном автоанализаторе ELISYS Uno (Human GmbH, Германия) и фотометре Stat Fax 303 (Awareness, США). В сыворотке крови определяли уровни: тиреотропного гормона (ТТГ), тироксина (Т4), свободного тироксина (свТ4), трийодтиронина (Т3), свободного трийодтиронина (свТ3) (ООО «Компания Алкор Био», Россия; Human GmbH, Германия), антител к тиреопероксидазе (АнтиТПО), антител к тироглобулину (АнтиТГ) (ООО «Компания Алкор Био», Россия; Euroimmun, Германия), тиреоглобулина (ТГ) (ООО «Компания Алкор Био», Россия; DRG Instruments GmbH, Германия), тиронинсвязывающего глобулина (ТСГ) (Monobit Ins, США). В плазме крови определяли уровни дофамина наборами фирмы Labor Diagnostika Nord (Германия). За норму принимались референсные значения, прилагаемые к используемым тест-наборам.

Для оценки функционального состояния щитовидной железы определялись универсальные системные индексы, позволяющие анализировать процессы периферической конверсии и механизмы обратной связи в системе «гипофиз — щитовидная железа»: индекс периферической конверсии (ИПК), индекс прогрессирующей периферической конверсии (ИпПК) и интегральный тиреоидный индекс (ИТИ) по следующим формулам:

ИПК = Т4 / ТЗ (1)

ИпПК = свТ4 / свТ3 (2)

ИТИ = (свТ3 + свТ4) / ТТГ (3)

## Статистический анализ

Проведена статистическая обработка данных лабораторных исследований с учетом пола (мужчины), группы населения (аборигенное население, местное европеоидное население), района проживания (г. Архангельск, поселки АЗРФ).

Статистическую обработку данных проводили с использованием пакета прикладных программ STATISTICA 10.0 (StatSoft, INC. USA, обладатель лицензии ФГБУН ФИЦКИА УрО РАН). Анализ соответствия вида распределения признака закону нормального распределения проводился с применением критерия Шапиро-Уилка. В связи с отклонением большинства изучаемых параметров от закона нормального распределения, применяли непараметрические критерии анализа. Описательная статистика количественных признаков представлена в виде центральной тенденции — медианы (Ме) и процентильных интервалов (10 и 90 процентилей). В тексте представлено как Ме (10; 90%). Проводился расчет значений доверительного интервала. Две независимые группы сравнивались с помощью U-критерия Манна-Уитни, три и более — с помощью рангового анализа вариаций по Краскелу-Уоллису с последующим парным сравнением групп тестом Манна-Уитни. Для оценки достоверности различий между связанными выборками применялся непараметрический дисперсионный анализ повторных измерений Фридмана с последующим попарным сравнением с помощью критерия Вилкоксона. Анализ различия частот в двух независимых группах проводился при помощи критерия χ² с поправкой Йетса. Для изучения связей между количественными показателями применяли ранговый коэффициент корреляции Спирмена.

## Этическая экспертиза

Комиссия по биомедицинской этике при Институте физиологии природных адаптаций УрО РАН постановила «принять положительное этическое заключение об одобрении пакета документов научного исследования согласно теме диссертационной работы» (протокол от 17.12.2010). Этический комитет ФГБУН ФИЦКИА РАН заключает: «Условия планируемых исследований соответствуют общепринятым нормам морали, соблюдены требования этических и правовых норм, а также прав, интересов и личного достоинства участников исследований; участники исследования информируются о целях, методах, ожидаемой пользе исследования; согласие субъекта на участие в исследованиях получено» (протокол № 2 от 04.11.2016).

## РЕЗУЛЬТАТЫ

В ходе аналитического поперечного неконтролируемого исследования было обследовано 120 мужчин, родившихся и постоянно проживающих на территориях АЗРФ: 20 человек в динамике четырех фотопериодов года (80 проб) — жители г. Архангельска в возрасте 22–44 лет (33,8±1,1 года (M±m)) — молодой возраст по классификации ВОЗ; 55 мужчин в возрасте 22–59 лет (44,6±1,6 года) — молодой и средний возраст по классификации ВОЗ, проживающих в поселках (русские — 28, оседлое аборигенное население — 18 и кочевое аборигенное население — 9 человек) — в период минимальной продолжительности светового дня; 45 мужчин в возрасте 22–59 лет (44,4±1,6 года), проживающих в поселках (русские — 30 и кочевые — 15 человек) — в период увеличения светового дня.

У жителей г. Архангельска вес, рост и ИМТ не изменялись по сезонам года и составили: 78,4±2,5 кг, 178,1±1,5 см, 24,8±0,9 кг/м².

У местных жителей г. Архангельска показано, что уровни дофамина в периферической крови были максимальны в июне и значимо снижались в период уменьшения продолжительности светового дня. Содержание дофамина в марте превышало его уровни в сентябре, а его медианные значения в марте и декабре не отличались. Концентрации, превышающие границу нормы, были выявлены у 15% (3/20 человека) в июне и по 5% (1/20) в марте и декабре. В сентябре высоких значений дофамина зарегистрировано не было. Недетектируемых значений дофамина (0 нмоль/л) в изучаемой группе также не наблюдали. При рассмотрении значений, отклоняющихся от 10 и 90 процентилей, было показано, что в декабре отмечается некоторый дисбаланс в содержании дофамина: так, у 15% (3/20) регистрировали повышенные, а у 20% (4/20) — пониженные значения.

Ранее нами была рассмотрена фотопериодическая динамика уровней тиреоидных гормонов и аутоантител [15], которая демонстрирует более низкие значения уровней общих фракций йодтиронинов и ТГ при повышении аутоантител к ТПО в осенне-зимний период. При анализе тиреоидных индексов, изменяющихся в динамике фотопериодов года, следует отметить ИПК, который повышался в осенний период относительно других сезонов. При отсутствии динамики свободных фракций йодтиронинов показана динамика уровней ИТИ, который в осенний период был максимален на фоне более низкого уровня ТТГ в крови. Содержание ТСГ в сыворотке крови было минимальным в декабре по сравнению с периодом максимального светового дня и его уменьшения (табл. 1).

**Table:** Таблица 1. Содержание гормонов системы «гипофиз — щитовидная железа», аутоантител к антигенам щитовидной железы дофамина в крови и показателей ССС у мужчин г. Архангельска в различные фотопериоды года (результаты представлены в виде медианы, 10; 90 процентилей и 95% доверительного интервала) Table 1. The content of hormones of the pituitary-thyroid system, autoantibodies to dopamine thyroid antigens in the blood and cardiovascular indicators in men in Arkhangelsk during various photoperiods of the year (results are presented as median, 10; 90 percentiles and 95% confidence interval)

Показатель	Март (1)	Июнь (2)	Сентябрь (3)	Декабрь (4)	Р-уровень
ТТГ 0,23–3,4 мМЕ/л	2,37 (0,87; 3,17) (1,80; 2,54)	2,01 (1,04; 4,06) (1,66; 2,55)	1,65 (0,86; 2,67) (1,43; 2,12)	2,56 (2,56; 3,59) (1,92; 2,90)	р2-3=0,007 р1-3=0,035 р3-4=0,026
Т3 1,0–2,8 нмоль/л	1,04 (0,86; 1,37) (0,98; 1,17)	1,09 (0,82; 1,38) (0,98; 1,19)	0,92 (0,73; 1,21) (0,85; 1,01)	0,94 (0,75; 1,26) (0,88; 1,07)	р1-3=0,004 р2-3=0,004 р2-4=0,003
Т4 53–158 нмоль/л	111,91 (95,96; 134,31) (107,13; 123,90)	113,45 (101,41; 126,49) (109,32; 118,40)	111,07 (87,22; 128,79) (102,13; 116,44)	99,03 (90,59; 117,13) (94,20; 107,92)	р1-4=0,002 р2-4=0,0002 р3-4=0,014
свТ3 2,5–7,5 пмоль/л	5,09 (4,16; 5,59) (4,75; 5,25)	5,21 (4,69; 6,68) (5,06; 5,71)	5,35 (4,39; 5,85) (4,82; 5,71)	5,23 (4,30; 6,07) (4,78; 5,50)	p>0,05
свТ4 10,0–23,2 пмоль/л	12,55 (11,20; 14,90) (12,24; 13,52)	13,05 (11,70; 14,90) (12,72; 13,80)	12,90 (10,30; 15,20) (11,78; 13,60)	12,90 (11,70; 15,10) (12,43; 13,72)	p>0,05
ИпПК (свТ4/свТ3), 1,37–4,43	2,59 (2,24; 3,28) (2,43; 2,78)	2,44 (2,10; 2,88) (2,35; 2,62)	2,44 (1,87; 3,10) (2,25; 2,67)	2,54 (2,16; 3,15) (2,39; 2,78)	p>0,05
ИТИ ((свТ4+свТ3)/ТТГ), 7,04–27,21	7,60 (4,61; 17,57) (7,05; 12,29)	9,18 (4,38; 15,72) (6,92; 15,86)	10,20 (4,11; 15,92) (8,96; 14,19)	7,33 (4,21; 16,24) (6,05; 13,91)	р1-3=0,021 р3-4=0,037
ИПК (Т4/Т3)	101,92 (91,13; 150,17) (99,58; 121,42)	106,54 (86,30; 146,81) (99,05; 117,19)	117,89 (94,96; 142,36) (110,91; 126,53)	102,89 (86,55; 129,41) (98,14; 113,31)	р2-3=0,006 р3-4=0,002
ТГ ≤55 нг/мл	26,91 (9,52; 52,61) (21,06; 36,28)	20,90 (20,90; 47,36) (18,24; 33,63)	20,21 (20,21; 47,64) (16,11; 29,58)	19,87 (19,87; 52,88) (15,79; 31,94)	р1-3=0,007 р1-4=0,018
ТСГ, 12–26 мкг/мл	13,98 (9,02; 18,46) (12,56; 15,57)	15,09 (10,82; 19,74) (13,76; 16,43)	14,62 (10,92; 19,13) (12,76; 16,20)	12,20 (8,03; 17,60) (11,09; 14,19)	р2-4=0,002 р3-4=0,019
АнтиТГ ≤65 Ед/мл	0,0 (0,0; 0,58) (0,027; 00,44)	0,0 (0,0; 0,29) (-0,026; 0,14)	0,0 (0,0; 0,87) (0,02; 0,39)	0,0 (0,0; 0,58) (-0,1; 0,51)	p>0,05
АнтиТПО ≤30 Ед/мл	0,08 (0,0; 2,59) (-0,30; 2,38)	0,24 (0,0; 5,99) (0,12; 2,77)	0,49 (0,08; 8,25) (-0,48; 5,82)	0,65 (0,0; 6,39) (0,07; 3,34)	р1-3=0,0005 р1-4=0,008
Дофамин (<0,653 нмоль/л)	0,365 (0,257; 0,555) (0,319; 0,438)	0,502 (0,324; 0,690) (0,431; 0,592)	0,235 (0,086; 0,455) (0,184; 0,308)	0,365 (0,018; 0,626) (0,256; 0,443)	р1-2=0,01 р1-3=0,008 р2-3=0,0003 р2-4=0,01
САД мм рт.ст.	127 (113; 140,5) (120,1; 137,5)	121,5 (115; 135) (119; 131,5)	122 (112; 155,5) (118,5; 137,5)	126,5 (107; 142,5) (119,8; 132,5)	p>0,05
ДАД мм рт.ст.	81 (65,5; 93,5) (75,4; 88,6)	77,5 (70,5; 93) (74,1; 84,3)	75 (64; 100,5) (72,9; 84,5)	77,5 (69,5; 90,5) (74,7; 83,9)	p>0,05
ЧСС уд/мин	66 (58,5; 84,0) (64,8; 73,3)	66 (56,5; 80,5) (63,1; 71)	71,5 (56,5; 80) (65,4; 74,6)	71,5 (59,5; 84,5) (67,4; 77,2)	p>0,05

Корреляции уровней дофамина с параметрами системы «гипофиз — щитовидная железа» показали, что максимальный уровень дофамина в июне имел связь с содержанием ТТГ (r=0,525; p=0,017), ИПК (r=-0,520; p=0,019) и ИпПК (r=-0,474; p=0,035). В период минимальной продолжительности светового дня регистрировали наличие связей между уровнями дофамина и Т3 (r=0,673; p=0,001), свТ3 (r=0,644; p=0,002), ИПК (r=-0,539; p=0,014). В этот же сезон года показана положительная связь между уровнем дофамина и АнтиТПО (r=0,771; p=0,00007). Корреляций между уровнем дофамина и показателями сердечно-сосудистой системы (САД, ДАД, ЧСС) выявлено не было.

Данные по антропометрическим показателям среди населения поселков и кочующего населения представлены в таблице 2. Анализ представленных данных позволил показать, что ИМТ практически не отличается у обследованных групп населения, при регистрации более низкого роста и веса у кочующего аборигенного населения по сравнению с местным европеоидным населением поселков.

**Table:** Таблица 2. Антропометрические данные у мужчин Арктической зоны РФ с учетом продолжительности светового дня и группы населения (результаты представлены в виде среднего значения (М) и стандартной ошибки среднего (m)) Table 2. Anthropometric data in men of the Arctic zone of the Russian Federation, taking into account the duration of daylight hours and population group (results are presented as the mean value (M) and standard error of the mean (m))

Показатель, ед. измерения	Период увеличения продолжительности светового дня(март)	Период минимальной продолжительности светового дня(декабрь)	р-уровень
кочующее аборигенное население	местное население поселков	кочующее аборигенное население	оседлое аборигенное население	местное население поселков
1	2	3	4	5
ИМТ, кг/м²	26,2±0,8	27,9±0,8	26,6±1,9	28,4±1,7	26,3±0,9	р>0,05
Вес, кг	73,0±2,4	84,1±2,2	68,3±5,0	81,1±4,8	80,8±4,3	р1-2=0,003
Рост, см	166±1,8	173,5±1,5	160,0±2,0	169±2,3	174,5±1,8	р1-2=0,006

При обследовании группы кочующего аборигенного населения было показано, что в период увеличения продолжительности светового дня по сравнению с минимальным световым днем ниже значение ИпПК, а также имеется тенденция более высокого содержания дофамина и ТГ при более низких значениях свТ4. Значения ЧСС при этом были выше у кочующего населения по сравнению с местным населением поселков. У местного европеоидного населения поселков, обследованного в эти же периоды, в марте было отмечено увеличение ИТИ при более низком содержании ТТГ и увеличении САД по сравнению с декабрем (табл. 3).

**Table:** Таблица 3. Содержание гормонов системы «гипофиз — щитовидная железа», аутоантител к антигенам щитовидной железы, дофамина в крови и показателей ССС у мужчин Арктической зоны РФ с учетом продолжительности светового дня и группы населения (результаты представлены в виде медианы, 10; 90 процентилей и 95% доверительного интервала) Table 3. The content of hormones of the pituitary-thyroid system, autoantibodies to thyroid antigens, dopamine in the blood and cardiovascular indicators in men in the Arctic zone of the Russian Federation, taking into account the length of daylight hours and the population group (results are presented as median, 10; 90 percentiles and 95% confidence interval)

Показатель, ед. измерения	Период увеличения продолжительности светового дня(март)	Период минимальной продолжительности светового дня(декабрь)	р-уровень
кочующее аборигенное население	местное население поселков	кочующее аборигенное население	оседлое аборигенное население	местное население поселков
1	2	3	4	5
ТТГ, мкМЕ/л	2,98 (1,55; 4,70) (1,41; 3,14)	1,60 (0,85; 3,50) (1,11; 1,87)	2,60 (0,60; 4,00) (0,71; 2,02)	2,10 (0,80; 5,30) (1,18; 2,36)	1,90 (1,30; 3,50) (0,65; 1,13)	р2-5=0,04 р1-2=0,005
Т3, нмоль/л	2,11 (1,39; 3,12) (0,60; 1,30)	1,71 (1,31; 2,18) (0,24; 0,41)	1,90 (1,10; 3,20) (0,52; 1,49)	1,45 (1,00; 1,90) (0,28; 0,56)	1,40 (1,10; 2,50) (0,46; 0,79)	р1-2=0,03
Т4, нмоль/л	95,96 (78,22; 113,29) (10,90; 24,22)	99,80 (68,30; 120,71) (14,71; 24,83)	111,50 (87,20; 126,00) (9,30; 26,38)	99,15 (73,50; 129,70) (19,64; 39,24)	105,60 (72,90; 126,50) (16,60; 28,58)	р>0,05
свТ3, пмоль/л	6,00 (5,30; 11,29) (1,76; 3,79)	5,70 (5,00; 6,60) (0,49; 0,84)	5,05 (1,50; 8,20) (1,52; 4,69)	4,70 (2,40; 8,50) (1,50; 3,07)	5,00 (3,40; 8,10) (1,63; 2,81)	р>0,05
свТ4, пмоль/л	13,90 (11,60; 16,00) (1,56; 3,36)	15,85 (12,40; 18,10) (1,78; 3,01)	15,20 (14,00; 18,10) (0,83; 2,38)	16,35 (11,90; 19,60) (2,07; 4,14)	16,25 (12,10; 19,60) (2,50; 4,30)	р1-3=0,015 р1-2=0,006
ИпПК свТ3/свТ4 усл. ед.	2,28 (1,19; 2,95) (0,54; 1,17)	2,77 (2,12; 3,48) (0,37; 0,63)	3,13 (1,85; 9,93) (1,98; 6,11)	2,95 (2,22; 6,35) (1,07; 2,19)	2,89 (1,73; 4,97) (1,44; 2,48)	р1-3=0,006 р1-2=0,003
ИТИ (свТ4+свТ3/ТТГ), усл. ед.	6,83 (4,27; 13,40) (3,05; 7,01)	14,33 (6,80; 30,13) (5,71; 11,19)	7,55 (5,50; 23,33) (4,16; 11,81)	10,89 (3,28; 27,89) (6,35; 12,69)	10,60 (5,63; 18,38) (3,24; 5,58)	р2-5=0,025 р1-2=0,002
ИПК (Т4/Т3), усл. ед.	45,64 (29,26; 64,37) (9,82; 21,82)	57,31 (43,17; 74,47) (10,60; 17,90)	49,16 (34,84; 114,55) (21,68; 61,50)	70,74 (49,00; 92,50) (13,31; 26,58)	58,01 (37,21; 104,58) (17,82; 30,69)	р1-2=0,009
ТГ, нг/мл	26,92 (11,12; 36,20) (9,29; 20,64)	6,57 (4,00; 23,23) (6,32; 11,13)	9,60 (2,00; 29,90) (6,45; 19,85)	5,00 (1,40; 11,60) (2,98; 6,11)	4,85 (1,00; 15,70) (5,32; 9,88)	р1-3=0,06 р1-2<0,001
АнтиТГ, МЕ/л	0,68 (0,00; 1,37) (0,52; 1,25)	-	0,20 (0,00; 9,00) (2,74; 7,79)	9,80 (6,90; 17,50) (2,81; 7,06)	8,90 (5,50; 20,80) (7,09; 14,17)	р3-40,003 р3-5<0,001
АнтиТПО, ME/мл	5,35 (0,89; 16,70) (5,82; 13,42)	-	2,75 (1,70; 20,20) (4,47; 13,74)	2,90 (1,60; 13,20) (3,5; 8,79)	3,35 (1,30; 14,70) (3,38; 6,76)	р>0,05
Дофамин, нмоль/л	0,394 (0,224; 0,788) (0,17; 0,38)	0,361 (0,204; 0,540) (0,11; 0,19)	0,00 (0,00; 0,914) (0,25; 0,70)	0,00 (0,00; 0,653) (0,22; 0,45)	0,248 (0,00; 0,849) (0,31; 0,54)	р1-3=0,07
САД мм рт.ст.	140,0 (129,0; 154,0) (133,7; 149,6)	145,0 (124,0; 170,0) (140,8; 155,1)	137,0 (125,0; 211,0) (41,9; 273,4)	134,0 (119,0; 161,0) (129,2; 146,5)	134,5 (118,0; 149,0) (127,8; 143,4)	р2-5=0,002
ДАД мм рт.ст.	93,0 (81,0; 111,0) (85,0; 100,1)	91,0 (76,0; 112,0) (85,6; 95,8)	93,0 (72,0; 102,0) (50,7; 127,2)	88,0 (71,0; 98,0) (79,8; 92,19)	86,5 (77,0; 94,0) (81,1; 88,8)	р>0,05
ЧСС уд/мин	82,0 (74,0; 103,0) (76,8; 90,8)	69,0 (56,0; 86,0) (65,6; 76,6)	72,5 (69,0; 76,0) (28,0; 116,9)	80,5 (66,5; 95,5) (71,2; 88,3)	74,0 (57,0; 89,0) (68,4; 77,9)	р1-2=0,008

Анализируя корреляции между изучаемыми параметрами для разных групп населения, выявили, что в период увеличения продолжительности светового дня связи между уровнем дофамина и общим Т3 (r=0,690; p=0,006) и свТ3 (r=0,672; p=0,009) характерны только для кочующего аборигенного населения. У местного европеоидного и оседлого аборигенного населения, соответственно, присутствуют положительные связи между содержанием дофамина и Т4 (r=0,445; p=0,016) и дофамина и АнтиТПО (r=0,670; p=0,025). У обобщенной выборки лиц, проживающих в поселках и кочующих, показано наличие корреляции между уровнем дофамина и САД (r= 0,26; p= 0,023).

Доля лиц с отклоняющимися от нормы значениями дофамина для разных групп населения поселков представлена в таблице 4. Больший процент обследуемых с недетектируемыми значениями дофамина (0 нмоль/л) выявлен в период минимальной продолжительности светового дня, что особенно выражено среди аборигенного населения, как кочующего, так и оседлого. Для кочующего и местного европеоидного населения показано наличие как недетектируемых, так и сверхнормативных значений дофамина. В период увеличения продолжительности светового дня для большой части кочующих аборигенов характерным является наличие сверхнормативных значений дофамина в отличие от европеоидного населения с референтными значениями дофамина.

**Table:** Таблица 4. Доля лиц (%) с различными уровнями дофамина в крови у мужчин Арктической зоны РФ с учетом продолжительности светового дня и группы населения Table 4. Proportion of individuals (%) with different levels of dopamine in the blood among men in the Arctic zone of the Russian Federation, taking into account the length of daylight hours and population group

Группа дофамина	Период увеличения продолжительности светового дня(март)	Период минимальной продолжительности светового дня(декабрь)	р-уровень
кочующее аборигенное население	местное население поселков	кочующее аборигенное население	оседлое аборигенное население	местное население поселков
1	2	3	4	5
Недетектируемый уровень дофамина (0 нмоль/л)	6	3	67	67	39	р1-3=0,0014 р2-5=0,0007
Референтный уровень дофамина (<0,653 нмоль/л)	67	94	11	28	39	р1-3=0,0077 р2-5<0,001
Сверхнормативный уровень дофамина (>0,653 нмоль/л)	27	3	22	5	22	р2-5=0,027

## ОБСУЖДЕНИЕ

## Репрезентативность выборок

Выборки обследуемых осуществлялись в определенные контрастные фотопериоды года среди мужчин, которые проживали либо в городе Архангельске, либо в поселках, ведя при этом кочевой, оседлый образ жизни (для аборигенов), или являлись местными неаборигенными жителями поселка, в результате чего полученные выборки являются репрезентативными для экстраполяции на целевые популяции мужчин.

## Сопоставление с другими публикациями

Данные по сезонному изменению уровней дофамина в периферической крови человека и животных практически отсутствуют [16], хотя фотопериодические эффекты на дофаминергичекие функции в головном мозге человека и животных изучались достаточно широко [17]. Полученные нами результаты по снижению уровней дофамина в периферической крови в период уменьшения продолжительности светового дня согласуются с исследованиями по изучению сезонных аффективных расстройств, которые проявляются в темный период года [18], и в механизмах развития которых значительную роль играет дофаминовая система головного мозга [19]. Максимальные значения дофамина регистрировали в июне, что согласуется с работами Timothy D. Brewertona [20], где было показано наличие положительных корреляций между содержанием в спинномозговой жидкости метаболита дофамина — гомованилиновой кислоты и солнечным светом у здоровых людей. В другом исследовании показатели гомованилиновой кислоты в спинномозговой жидкости здоровых людей были самыми низкими весной и самыми высокими летом [21]. Есть мнение, что, несмотря на разные источники синтеза центрального и периферического дофамина, они могут иметь общую регуляцию, хотя находятся под контролем со стороны различных промоторных областей [22]. Кроме того, по некоторым данным, дофамин головного мозга может влиять на уровень периферического дофамина [23]. Периферический дофамин, который мы рассматриваем в своей работе, оказывает влияние на сердечно-сосудистую, пищеварительную, иммунную и гормональную системы, а также регулирует функцию почек [22]. Вследствие этого знание фотопериодической динамики уровня плазменного дофамина является новым и значимым фактом не только для фундаментальной физиологии, но и для практического здравоохранения.

Рассматривая соотношение уровней периферического дофамина и показателей гипоталамо-гипофизарно-тиреоидной системы у жителей г. Архангельска, обнаружено, что максимальные значения дофамина в июне выявлены на фоне более высокой активности системы «гипофиз — щитовидная железа» (Т3, Т4) с регистрацией отрицательной корреляции дофамина с ИПК, что, возможно, указывает на стимулирующую роль дофамина в процессах конверсии тиреоидных гормонов, механизмах их синтеза и секреции, что ранее было показано в экспериментальных работах [14][24].

Снижение уровня дофамина в период уменьшения светового дня сочетается с понижением периферической конверсии йодтиронинов, что подтверждает положение о влиянии дофамина на механизмы конверсии тиреоидных гормонов. Соответственно, в этот период при неизменном уровне общего Т4 значимо снижается содержание общих фракций более активного Т3. Кроме того, снижается уровень ТГ — белка, выполняющего важную роль в синтезе тиреоидных гормонов и являющегося резервом йодтиронинов в полости фолликула. Это косвенным образом может указывать на замедление процессов синтеза и запасания тиреоидных гормонов в тиреоцитах, т.к. небольшое количество ТГ высвобождается из тиреоцитов в кровоток [25–27]. При этом известно о роли дофамина в поглощении йода и его органификации, что также усиливает процессы синтеза тиреоидных гормонов, а также о значении дофамина в индукции секреции гормонов щитовидной железы путем эндоцитоза тиреоглобулина и выброса тиреоидных гормонов в кровь [24]. Снижение уровня дофамина в осенний период сопряжено с нарастанием уровней АнтиТПО, что может объясняться снижением ингибирующего воздействия высоких уровней дофамина на антителообразование, в частности на продукцию иммуноглобулина класса G, к которым относится АнтиТПО. Одним из возможных механизмов влияния дофамина на иммунную систему является стимулирование лимфопролиферации и активизации дифференцировки Т-хелперов, что в итоге приводит к повышению созревания антителообразующих клеток и синтезу иммуноглобулинов. Однако было показано, что при высоком уровне дофамина регистрировалось повышение содержания иммуноглобулинов классов IgM и IgE и снижение уровней IgG и IgA [28–30]. В свою очередь повышение уровней АнтиТПО может способствовать угнетению процесса синтеза тиреоидных гормонов, т.к. тиреопероксидаза участвует в механизмах продукции тиреоидных гормонов. Таким образом, сочетание низких уровней дофамина с уменьшением значений ИПК, ТГ и повышением АнтиТПО в сентябре, возможно, указывает на снижение дофаминовой регуляции аутоиммунных реакций, а также процессов конверсии тиреоидных гормонов, их синтеза и запасания в этот период года.

В периоды минимальной продолжительности и увеличения продолжительности светового дня показаны сходные уровни дофамина, однако активность щитовидной железы выше в период увеличения светового дня (Т4, ТГ) при более низком содержании АнтиТПО. Максимальный уровень АнтиТПО в декабре, которые, нейтрализуя тиреоидную пероксидазу, могут снижать синтез йодтиронинов, сочетается с минимальными значениями тиреоидных гормонов, что, возможно, свидетельствует о роли антитиреоидных аутоантител в регуляции функции щитовидной железы в этот период. Однако в декабре отмечается также большое количество корреляций между содержанием дофамина, йодтиронинами и ИПК, вероятно, указывающих на его значимость в регуляции их содержания в крови, что объясняется физиологической связью между дофаминергической и тиреоидной системами. Кроме того, по результатам корреляционного анализа можно предположить, что стимулирующее влияние дофамина на периферическое превращение тиреоидных гормонов наиболее ярко проявляется в контрастные фотопериоды года — максимальной и минимальной продолжительности светового дня.

Среди жителей поселков АЗРФ, обследованных в период увеличения продолжительности светового дня и его минимальной продолжительности, наибольшая реактивность со стороны изучаемых систем на изменение фотопериода года выявлена у кочующего аборигенного населения, что проявляется тенденцией нарастания уровней дофамина и ТГ и снижении ИпПК и свТ4 в марте, а также недетектируемыми значениями дофамина при более низкой активности гипоталамо-гипофизарно-тиреоидной системы в декабре. Кроме того, у кочующего населения в марте регистрируются положительные взаимосвязи между уровнями дофамина и содержанием общих и свободных фракций Т3, что может предполагать их взаимный модулирующий эффект. Следовательно, у кочующего населения показано однонаправленное нарастание уровней дофамина и активности системы «гипофиз — щитовидная железа» в период увеличения продолжительности светового дня по сравнению с периодом минимального светового дня.

Местное европеоидное население поселков, вблизи которых кочевало аборигенное население, при отсутствии фотопериодической динамики уровней дофамина имеет реакции со стороны ТТГ на изменение фотопериодов года, что, возможно, связано с температурным фактором. Схожие реакции наблюдались у жителей г. Архангельска, у которых в марте по сравнению с декабрем отсутствовала динамика уровней дофамина при снижении АнтиТПО и нарастании Т4. Вероятно, в период увеличения светового дня, когда значительно меняется освещенность, а не температура, роль дофамина в регуляции активности гипоталамо-гипофизарно-тиреоидной системы у местных жителей Европейского Севера снижается, в то время как у кочующих аборигенов — усиливается. Ранее исследователями были показаны более яркие изменения со стороны свТ4 и ТТГ в зимний период года по сравнению с летним у якутских мужчин, ведущих традиционный образ жизни [31].

Полученные результаты еще раз подчеркивают большую чувствительность адаптационных систем организма и их взаимодействие при изменении климато-географических условий у коренных народов.

Особо хочется отметить, что дисбаланс содержания дофамина чаще выявлялся у жителей поселков, чем у жителей г. Архангельска. Большой процент лиц с недетектируемыми значениями дофамина (0 нмоль/л) в период минимального светового дня, характерный в большей степени для кочевых и оседлых аборигенов, может являться как предиктором развития депрессивно-подобных отклонений, связанных с сезонным фактором [18][19][32], так и предпосылкой снижения активности щитовидной железы ввиду стимулирующего влияния референтных значений дофамина на продукцию йодтиронинов [24][33]. У жителей г. Архангельска в декабре также наблюдали некоторый дисбаланс в содержании дофамина, который, однако, не выходил за границы нормы. Кроме того, параллельное снижение уровней дофамина и гормонов щитовидной железы наблюдали также в период уменьшения продолжительности светового дня у местного русского населения г. Архангельска. Кроме низких значений дофамина в декабре, регистрировались также сверхнормативные значения дофамина среди кочующего и европеоидного населения, а также у кочующего населения в период увеличения продолжительности светового дня, что может указывать на риск развития сердечно-сосудистых заболеваний у лиц данных групп. Особому напряжению подвергается ССС в период увеличения продолжительности светового дня при нарастании показателей САД, а также в свете выявления положительной корреляции между уровнем дофамина и САД. Выявленная ситуация указывает на необходимость проведения диагностических и профилактических мер у населения Европейского Севера в периоды уменьшения и минимальной продолжительности светового дня, а также при его нарастании, и дополнительных исследований, выявляющих возможные климато-географические, социально-экономические и психологические факторы этого явления.

Выявленное нами снижение активности дофаминергической и гипоталамо-гипофизарно-тиреоидной систем в периоды уменьшения и минимальной продолжительности светового дня (сентябрь, декабрь) и повышение их активности в периоды нарастания и максимальной длительности светового дня (март, июнь) может быть связано с ингибирующей ролью мелатонина в отношении щитовидной железы и симпатической нервной системы в темные фотопериоды года. В защиту данной гипотезы можно привести сведения о выявленной фотопериодической динамике мелатонина [34] и его роли в регуляции функции щитовидной железы [1] и дофаминергической системы [10]. Снижение активности симпатоадреналовой системы и щитовидной железы может приводить к замедлению активности обменных процессов в этот световой период, что может сопровождаться нарастанием массы тела и является критерием риска развития ожирения и сахарного диабета.

Следовательно, нами показаны критерии риска изменения активности дофаминергической и тиреоидной систем у разных групп населения Европейского Севера с учетом фотопериодов года:

## Клиническая значимость результатов

Ввиду большой значимости периферического дофамина в регуляции всех функций организма, сведения о его фотопериодической динамике, а также возможном влиянии на активность щитовидной железы у разных групп населения Европейского Севера могут помочь в организации диагностических, профилактических и лечебных мероприятий, направленных на стабилизацию уровней дофамина, йодтиронинов и аутоантител к антигенам щитовидной железы с учетом фотопериода и группы обследованных. В целом при низкой активности щитовидной железы необходимо рекомендовать обследование на содержание уровня дофамина в крови.

Одними из способов повышения уровней дофамина и активности щитовидной железы в неблагоприятные периоды года (снижение продолжительности светового дня и минимальной его продолжительности) являются: коррекция питания с употреблением в пищу продуктов, содержащих тирозин, фенилаланин, употребление препаратов, содержащих широкий спектр витаминов и микроэлементов, участвующих в синтезе гормонов щитовидной железы, соблюдение рационального режима труда и отдыха, включающего в себя полноценный сон, оптимальный двигательный режим, пребывание на свежем воздухе, активный и пассивный отдых и т.д., нормализация массы тела. С целью повышения активности дофаминергической системы и щитовидной железы в неблагоприятные периоды года целесообразно использовать немедикаментозные средства коррекции. В случае отсутствия эффекта профилактических мероприятий, направленных на нормализацию уровня дофамина в крови, рекомендуется обращение к узкопрофильным специалистам (психолог, психотерапевт) для подбора корректных методов устранения дефицита уровня дофамина. Возможное снижение активности обменных процессов в этот период года обуславливает необходимость проведения контроля массы тела и уровня сахара в крови. Кроме того, указанные фотопериоды являются наиболее критическими в плане аутоиммунной составляющей функции щитовидной железы, что предполагает проведение диагностики уровней АнтиТПО в выявленные периоды года.

Выявлено, что у жителей поселков регистрируется зависимость САД от уровня дофамина в крови и высокие концентрации дофамина показаны у них как в период минимального светового дня, так и в период его увеличения. Следовательно, данные группы обследованных более подвержены заболеваниям со стороны ССС, ввиду чего рекомендуется проводить как самостоятельный мониторинг САД, ДАД, ЧСС, так и мониторинг функционирования ССС в лечебных учреждениях и, в случае выявления отклонений от нормативных показателей, обращаться к кардиологу.

## Ограничения исследования

Ограничением исследования стало обследование населения поселков (местное европеоидное население и оседлые аборигены), а также кочующих аборигенов, которые кочевали близ этих поселков только в течение двух фотопериодов года, когда была возможность обследования нескольких групп населения одновременно для проведения сравнительного анализа. Однако в период увеличения продолжительности светового дня в обследуемых поселках, вблизи которых в марте кочуют оленеводы, проживает лишь местное европеоидное население, в связи с чем не удалось обследовать оседлое аборигенное население. Кроме того, одним из ограничений является обследование лишь группы мужского населения с целью получения первичных сведений, которые впоследствии будут сравниваться с данными женской популяции. В связи с этим результаты и выводы представлены лишь по имеющимся фактическим данным с некоторыми ограничениями.

## Направления дальнейших исследований

Продолжением исследования может служить выявление фотопериодической динамики уровней дофамина крови и гормонов щитовидной железы у женского населения Европейского Севера, а также изучение возможного влияния дофамина на другие эндокринные звенья, в частности, систему «гипоталамус — гипофиз — гонады».

## ЗАКЛЮЧЕНИЕ

Показаны однонаправленные изменения в крови уровней дофамина и гормонов щитовидной железы в различные фотопериоды года у жителей Европейского Севера со снижением активности дофаминергической и гипоталамо-гипофизарно-тиреоидной систем в периоды уменьшения и минимальной продолжительности светового дня (сентябрь, декабрь) и повышением их активности в периоды нарастания и максимальной длительности светового дня (март, июнь). Для кочующего аборигенного населения значимыми периодами для однонаправленного изменения активности дофаминергической системы и щитовидной железы являются периоды минимального светового дня и увеличения продолжительности светового дня, в то время как у местного европеоидного населения поселков такая динамика отсутствовала. У местного европеоидного населения г. Архангельска однонаправленные изменения изучаемых систем регистрировали в периоды снижения и максимальной продолжительности светового дня. Аборигенное и европеоидное население поселков имеет выраженный дисбаланс содержания дофамина в период минимального светового дня по сравнению с европеоидным населением г. Архангельска.

## ДОПОЛНИТЕЛЬНАЯ ИНФОРМАЦИЯ

Источники финансирования. Исследование выполнено за счет гранта Российского научного фонда №23-25-10027, а также финансового обеспечения Минобрнауки РФ (гос. задание ФГБУН ФИЦКИА УрО РАН; номер гос. регистрации 122011800392-3).

Конфликт интересов. Авторы декларируют отсутствие явных и потенциальных конфликтов интересов, связанных с содержанием настоящей статьи.

Участие авторов. Типисова Е.В. — концепция, дизайн, получение, анализ и интерпретация результатов, написание статьи; Зябишева В.Н. — получение данных, написание статьи; Аликина В.А. — получение данных, написание статьи; Елфимова А.Э. — получение данных, внесение в рукопись существенной правки с целью повышения научной ценности статьи; Молодовская И.Н. — получение данных, написание статьи. Все авторы одобрили финальную версию статьи перед публикацией, выразили согласие нести ответственность за все аспекты работы, подразумевающую надлежащее изучение и решение вопросов, связанных с точностью или добросовестностью любой части работы.

Благодарности. Авторы благодарят сотрудников Центра профессиональной диагностики ООО «Биолам» за забор крови у обследованных лиц г. Архангельска и медицинский персонал, проводивший забор крови в экспедициях.
